# Tracing energy inputs into the seafloor using carbonate sediments

**DOI:** 10.1073/pnas.2215833120

**Published:** 2023-02-21

**Authors:** B. P. Smith, S. M. Edie, W. W. Fischer

**Affiliations:** ^a^Division of Geological and Planetary Sciences, California Institute of Technology, Pasadena, CA 91125; ^b^Department of Paleobiology, National Museum of Natural History, Smithsonian Institution, Washington, D.C. 20013

**Keywords:** bioturbation, end-Permian extinction, seafloor energetics, ocean chemistry, diagenesis

## Abstract

Carbonate rocks provide unique and valuable sedimentary archives for secular changes in Earth’s physical, chemical, and biological processes. However, reading the stratigraphic record produces overlapping, nonunique interpretations that stem from the difficulty in directly comparing competing biological, physical, or chemical mechanisms within a common quantitative framework. We built a mathematical model that decomposes these processes and casts the marine carbonate record in terms of energy fluxes across the sediment–water interface. Results showed that physical, chemical, and biological energy terms across the seafloor are subequal and that the energetic dominance of different processes varies both as a function of environment (e.g., onshore vs. offshore) as well as with time-varying changes in seawater chemistry and with evolutionary changes in animal abundance and behavior. We applied our model to observations from the end-Permian mass extinction—a massive upheaval in ocean chemistry and biology—revealing an energetic equivalence between two hypothesized drivers of changing carbonate environments: a reduction in physical bioturbation increased carbonate saturation states in the oceans. Early Triassic occurrences of ‘anachronistic’ carbonates—facies largely absent from marine environments after the Early Paleozoic—were likely driven more by reduction in animal biomass than by repeated perturbations to seawater chemistry. This analysis highlighted the importance of animals and their evolutionary history in physically shaping patterns in the sedimentary record via their impact on the energetics of marine environments.

## Significance

The seafloor represents a critical boundary between Earth’s interior and its surface. Any process that transports mass or energy across this boundary helps regulate Earth’s oceans, atmosphere, and biosphere—both at present and in the past. While geologists and geochemists often use principles of mass transfer, fluxes of energy across the seafloor have proven difficult to constrain. We developed a quantitative framework for energy transfer across the seafloor that integrates physical, chemical, and biological processes. We then use our framework to understand how changes in carbonate sediments through time reflect two major drivers in our planet’s history: changes in oceanic chemistry and evolutionary innovations in the history of life (e.g., burrowing behaviors).

Marine carbonate rocks provide important records of Earth’s physical, chemical, and biological history (1–3). Because carbonate minerals dissolve and precipitate quickly at the Earth’s surface conditions, they often contain physical (4–7) and chemical (8–11) records of near-surface environments across local, regional, and global scales. Marine carbonates also undergo sediment transport, making bedforms that preserve a history of physical ocean conditions (12, 13). Carbonate sediments are among the largest fluxes in Earth’s carbon cycle, constituting a key removal pathway for atmospheric CO_2_ over geologic timescales (14, 15). Additionally, carbonates are tied to many other biogeochemical cycles by way of microbial metabolisms (16). The sensitivity of carbonates and their deposits to each of these processes makes them useful as historical archives, but interactions among these processes can lead to conflicting or nonunique interpretations (17–19).

The secular trend in the types and frequency of carbonate facies from the Proterozoic to today has been attributed to multiple factors, including global changes in ocean carbonate chemistry (2, 3, 17, 20, 21) and the evolution of animals (22–26). Both factors were likely involved, but their relative contributions can be difficult to model from the characteristics of the facies alone. For example, carbonate facies from the early Paleozoic often contain “flat-pebble conglomerate” deposits, which are the products of storm events that ripped up and redeposited early cemented substrates. These textures largely disappeared following the Ordovician radiation of burrowing animals, suggesting that the physical disruption from bioturbation “outcompetes” cementation and thus the formation of these cemented clasts (23). The reemergence of flat-pebble conglomerates in the wake of the end-Permian mass extinction suggests that when animal diversity and abundance decline to early Paleozoic levels, then early cementation becomes more prominent on the seafloor (27). However, elevated carbonate saturation states and widespread anoxia in the earliest Triassic oceans may also account for the reemergence of flat-pebble conglomerates (28, 29), an interpretation which emphasizes the role of burrowing organisms in regulating porewater chemistry near the sediment–water interface (30–33). Modeling the relative inputs of the biological and chemical factors in a common framework can help to constrain the underlying drivers for “anachronistic” facies in the Permian-Triassic and throughout Phanerozoic time.

**Fig. 1.**
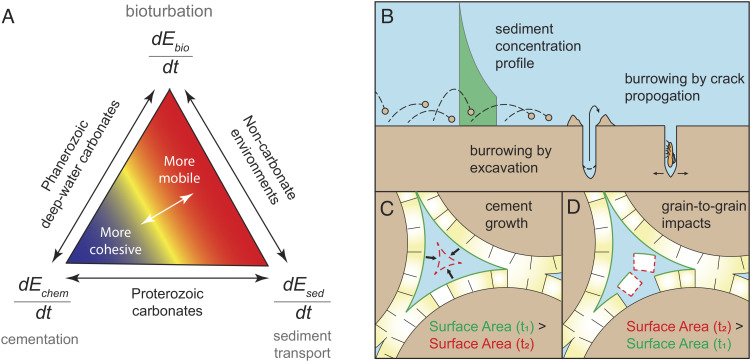
Factors that promote or hinder cohesion within carbonate substrates. (*A*) Ternary diagram to illustrate how chemical, physical, and biological factors affect carbonate cementation. The labeled derivative at each point of the triangle corresponds to an energy flux in Eqs. 2–4. The lower left vertex represents favorable chemical conditions in seawater or within pore spaces that promote high rates of carbonate cementation and thus substrate cohesion. This process is counteracted by burrowing animals (*Top* vertex: “bioturbation”) and/or by sediment transport (*LowerRight* vertex), both of which expend metabolic energy to break up nascent cements and mobilize substrata. (*B*) Cross-section of the seafloor showing physical factors that transfer energy across the sediment–water interface. During sediment transport, grains distributed throughout the water column (green curve) episodically strike the bed, transferring energy from the flow. Burrowing animals also transfer energy, either by excavating sediment or by crack propagation. (*C*) How mineral surface area changes during cementation. As cementation progresses, the surface area at some later time (*A*_*t*_2__) is less than the initial surface area (*A*_*t*_1__). (*D*) How physical energy fluxes act to increase surface area. Energy fluxes from burrowing animals or from sediment transport knock off nascent cements so that the surface area at a later time (*A*_*t*_2__) is greater than at an earlier time (*A*_*t*_1__).

We analyzed the relative inputs of chemical, physical, and biological factors acting on the seafloor in an order-of-magnitude, energy-based framework. This model sums contributions from physical, chemical, and biological factors—namely transport conditions, seawater chemistry, and animal size and behavior—and permits direct comparison of competing mechanisms (Fig. 1*A*). The model revealed that energies associated with these processes are subequal and that variations across time and space–particularly those related to changing ocean chemistry or animal evolution and ecology–can greatly help or hinder the formation of lithified substrates. We then used our model to show how these solution spaces can be used to integrate and compare diverse factors that affect carbonate cementation after the end-Permian extinction, the most extreme, natural reorganization of marine ecosystems and seawater chemistry during the Phanerozoic eon.

## Modeling Energetics of Seafloor Cohesion

Three components that influence sediment cohesion were quantified here: carbonate mineral precipitation, sediment transport, and bioturbation. Each component was modeled as an energy flux expressed in Joules per unit area per unit time [J/m^2^s]. The expressions developed below and in *Materials and Methods* relate the energy fluxes of components to each other through the physical and chemical properties of the environment and through the population-level bioturbation dynamics of certain marine organisms.

For a given volume of sediment and fluid, a portion of the Gibbs free energy, known as interfacial free energy (*G*_*int*_), is bound at the contacts between crystals and their surrounding fluid. The rate of change $\frac{dG_{\mathrm{int}}}{\mathrm{dt}}$ is proportional to the change in the mineral surface area:

[1] $$\begin{array}{c} \frac{dG_{\mathrm{int}}}{\mathrm{dt}}=\sigma \frac{\mathrm{dA}}{\mathrm{dt}}, \end{array}$$

where *σ* is a constant representing the specific free energy for the mineral–fluid contact (15). Following ref. 27, we cast $\frac{dG_{\mathrm{int}}}{\mathrm{dt}}$ as a competition between chemical and physical processes that change the mineral surface area, *A*, and by extension the interfacial energy, G_*int*_ (Fig. 1*A*). Thus, the energy balance for this competition is

[2] $$\begin{array}{c} \frac{dG_{\mathrm{int}}}{\mathrm{dt}}=\frac{dE_{\mathrm{sed}}}{\mathrm{dt}}+\frac{dE_{\mathrm{bio}}}{\mathrm{dt}}-\frac{dE_{\mathrm{chem}}}{\mathrm{dt}}, \end{array}$$

where $\frac{dE_{\mathrm{sed}}}{\mathrm{dt}}$ and $\frac{dE_{\mathrm{bio}}}{\mathrm{dt}}$ are the physical energy fluxes contributed by sediment transport and burrowing animals, respectively, and $\frac{dE_{\mathrm{chem}}}{\mathrm{dt}}$ is the change in interfacial energy during cementation (Fig. 1). Note that $\frac{dE_{\mathrm{chem}}}{\mathrm{dt}}$ is negative because cementation decreases available surface area, acting against the two physical energy terms that fracture grains and increase surface area (Fig. 1 *C* and *D*). Thus, the sign and magnitude of Eq. **2** express the likelihood that carbonate sediments will develop features associated with early cementation. If the physical energy terms ($\frac{dE_{\mathrm{sed}}}{\mathrm{dt}}$ or $\frac{dE_{\mathrm{bio}}}{\mathrm{dt}}$) exceed $\frac{dE_{\mathrm{chem}}}{\mathrm{dt}}$, then $\frac{dG_{\mathrm{int}}}{\mathrm{dt}}$ is positive and kinetic energy can dislodge nascent marine cements, preventing cements from forming cohesive substrates and their associated sedimentary structures. In contrast, if $\frac{dE_{\mathrm{chem}}}{\mathrm{dt}}$ exceeds the summed physical energy terms, then cementation occurs and, over time, individual grains will form aggregates or cohesive substrates.

### Porewater Chemistry and Interfacial Free Energy.

The growth of authigenic minerals primarily promotes substrate cohesion in carbonate sediments. In terms of energy flux, the rate of energy change to the supersaturation of the fluid, *Ω*, depends on mineralogy and the initial specific surface area, A_*sp*_:

[3] $$\begin{array}{c} \frac{dE_{\mathrm{chem}}}{\mathrm{dt}}\approx -\frac{2}{3}\sigma \frac{{\left(A_{\mathrm{sp}}\left(1-\phi_{0}\right)\right)}^{2}}{\phi_{0}}\rho_{c}M_{c}k{\left(\Omega -1\right)}^{n}*L, \end{array}$$

where *ϕ*_0_ is the initial porosity; *ρ*_*c*_ and *M*_*c*_ are the density and molar mass of the carbonate phase; *k* and *n* are constants associated with a kinetic rate law for precipitation; and the vertical length scale of the observation window within the sediment, *L*, is assumed to be the depth of active bioturbation. The units, values, and sources for these constants are listed in Table 1. The power law dependence on *Ω* is a typical formulation from carbonate precipitation and dissolution experiments (34).

**Table 1.** Model parameters and constants

Parameter	Value	Units	Source
*k*	0.113	mol m^2^ yr^-1^	refs. 5, 34
*n*	2.26	-	refs. 5, 34
*M*_*a* *r* *a* *g*_	0.1	kg mol^-1^	-
*ϕ*	0.4	-	-
*ρ*_*a* *r* *a* *g*_	2,850	kg m^3^	-
*σ*	0.283	J m^-2^	ref. 40
*A*_1_	0.3	-	ref. 36

The initial specific surface area, A_*sp*_, strongly depends on the grain size. Although reactive surface areas in carbonates are a complex function of grain shape and microstructure, ref. 35 proposed an approximation based on dissolution experiments:

[4] $$\begin{array}{c} A_{\mathrm{sp}}=\left(\frac{1}{\rho_{c}D}\right)r_{f}. \end{array}$$

The bracketed term in Eq. **4** is a simple geometric model in which the surface area-to-volume ratio scales inversely with grain diameter, D. The term r_*f*_ is a roughness factor, representing additional surface area due to microscale roughness. Ref. 35 suggests that a roughness factor of five is appropriate for both shallow- and deep-water carbonate sediments. Although Eq. **4** is approximate, it provides an explicit link to the grain diameter, D, which creates a conceptual link with other sedimentary processes that depend on grain size.

### Energy Transfer During Sediment Transport.

During transport, sediments vacillate between entrainment in the flow and deposition on the bed. When particles strike the bed, they impart kinetic energy at rates that scale with impact velocity and frequency. Calculating the rate of kinetic energy transfer is important for solving several problems in sedimentology and geomorphology such as the rate of bedrock erosion in rivers (36) and the relationship between ooid size and local environmental conditions (5). We modified an erosion model from ref. 7 by truncating their calculation at the penultimate step to show energy transfer rather than erosion/abrasion rate (*Materials and Methods*). The resulting expression is

[5] $$\begin{array}{c} \frac{dE_{\mathrm{sed}}}{\mathrm{dt}}=\frac{1}{2}\frac{A_{1}\rho_{c}qw_{i}^{3}}{UH\chi +U_{b}H_{b}}, \end{array}$$

where A_1_ is an empirically determined dimensionless constant, *ρ*_*c*_ is the density of carbonate, *q* is the rate of sediment supply, *w*_*i*_ is the impact velocity normal to the bed, *H*_*b*_ and *U*_*b*_ are the average height and velocity of the bedload layer, *U* is the total depth-averaged velocity, *H* is the flow depth, and *χ* is an integral that described the vertical profiles of velocity and sediment concentration in the flow.

When kinetic energy from impacts passes into the bed, it can increase *ΔG*_*int*_ by fracturing grains. While this process is not the only way energy can be dissipated in the bed, the total kinetic energy transfer rate in Eq. **5** nevertheless provides an upper limit on maximum energy available to create new surfaces through sediment transport.

### Energy Transfer from Burrowing Animals.

The third component of the energy balance framework considers animal interactions with the sediment, which can be both physical and chemical in nature. Animals can irrigate porewaters with oxygenated waters, which lowers the saturation state (*Ω* in Eq. **3**) by promoting aerobic respiration (31) and thus reducing the cementation rate. However, we focused here on the physical transfer of energy from animals into sediments through their burrowing or excavation behaviors, a portion of which can be used to grind or break up cements (Fig. 1*B* –*D*). As for the energy flux from sediment transport, this bioturbation process increases the surface area of mineral–water contacts, which counteracts cementation.

In practice, bioturbation is a catch-all term for a wide array of locomotion and/or feeding strategies that have different effects on sediment mixing. Many classification schemes (e.g., ref. 37) divide organisms into functional groups based on how they interact with sediments rather than the identity of species present. While a full analysis of all functional groups is beyond the scope of this work, we focused on two cases that served as useful endmembers. One such functional group, upward conveyors, transports particles from the bottom of the mixed layer to the surface. At a minimum, we can say that the energy flux across the sediment–water interface must account for the change in gravitational energy of the particles:

[6] $$\begin{array}{c} \frac{dE_{\mathrm{bio}}}{\mathrm{dt}}=\frac{dE_{\mathrm{gr}}}{\mathrm{dt}}=\frac{1}{2}\left(1-\phi \right)\left(\rho_{s}-\rho_{w}\right)Rgd, \end{array}$$

where *g* is gravitational acceleration, *ρ*_*s*_ and *ρ*_*w*_ are the densities of sediment and water, respectively; *R* is the volumetric rate at which animals burrow, *ϕ* is porosity; and *d* is the depth of the burrow.

Once sediments become cohesive, individual particles become more difficult to move. Some organisms that inhabit muddy substrates, such as polychaetes, burrow by propagating a fracture tip through the sediments. Following (38), the energy flux from burrowing, $\frac{dE_{\mathrm{cr}}}{\mathrm{dt}}$, can be modeled as

[7] $$\begin{array}{c} \frac{dE_{\mathrm{bio}}}{\mathrm{dt}}=\frac{dE_{\mathrm{cr}}}{\mathrm{dt}}=\frac{2G_{c}R}{\pi b}, \end{array}$$

where G_*c*_ is the fracture toughness and *b* is the width of the burrow.

Although the two burrowing methods outlined above do not capture the breadth of real marine systems, they likely represent useful end members near the upper and lower bounds of energy expenditure. The energy flux from excavation likely provides a lower bound because it does not account for other kinds of particle displacement, such as diffusion, nor does it account for particle rotation. In contrast, the crack-propagation model likely represents an upper limit as it requires an organism to overcome the fracture toughness of the substrate. If the fracture toughness is too high—for example, in carbonate-cemented hardgrounds—organisms likely resort to strategies such as boring by chemical dissolution rather than burrowing (39).

Despite their simplicity, both models of bioturbation energy flux allow animal ecology and population to be expressed in terms of a volumetric rate $\frac{\mathrm{dV}}{\mathrm{dt}}$ that animals rework sediment. Empirically derived sediment reworking rates for different taxonomic groups that vary in body size, sediment interaction behavior, and population density (22) were used to determine a range of bioturbation energy fluxes. Many of the trends in bioturbation discussed in ref. 22 can be related directly to the energy output of certain taxa because Eqs. **6** and **7** linearly depend on the volumetric sediment reworking rate *R*.

## Results

We considered bivariate interactions of the fluxes in the energy balance equation, Eq. **2** to analyze endmember pairs of processes, i.e., the edges of the conceptual ternary diagram in Fig. 1*A*. These edge cases are often used to interpret the mechanisms behind the development of certain sedimentary fabrics and facies in the rock record. For example, during the evolution of early marine animals, carbonate substrate cohesion would have been primarily a function of sediment transport and cementation, which likely underlies the formation of prominent Paleozoic carbonates such as flat pebble conglomerates. However, as marine animals diversified and bioturbation intensified, sediments mobilized in regions with typically low physical transport (e.g., deep-water settings; Fig. 1*A*) (41). Ultimately, all three energy fluxes are relevant for the long- and short-term reactivity and stability of carbonate sediments.

### Quiet-Water Environments: Bioturbation vs. Cementation.

**Fig. 2.**
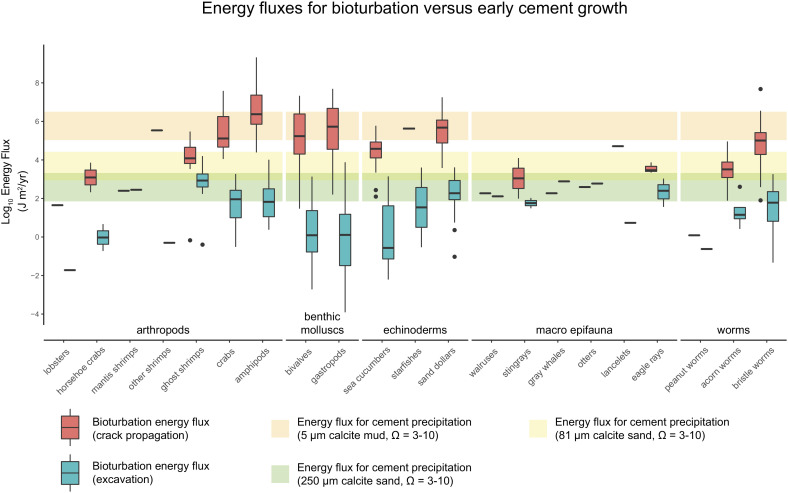
Typical ranges for chemical energy transfer rates in carbonate-forming environments and bioturbation energy transfer rates calculated from ref. 22. Box plots show physical transfer of energy during bioturbation calculated using both the crack-propagation model in dark blue, Eq. **7** and the excavation model in light blue, Eq. **6**. Box plots are grouped by rough taxonomic groups. The red, yellow, and green bands show ranges of energy transfer during cementation and reflect different combinations of porewater chemistry (i.e., calcite saturation state), and grain size, which determines initial surface area, Eq. **3**. The large degree of overlap between the box plots and bands implies that the relative contributions that the dominance of either chemical or biological activity–and thus, the presence of early cemented features–can be altered by changing any combination of animal ecology, sediment size, and porewater chemistry.

In transport conditions below, the threshold of motion for sediments, substrate cohesion reflects a competition between bioturbation and cementation energy fluxes (Fig. 2). In marine environments, this case reflects deeper-water settings with lower shear velocities and intermittent sediment transport during storms (Fig. 1*A*).

The energy-transfer rates in Fig. 2 vary over almost ten orders of magnitude, varying with changes in animal ecology, porewater chemistry, and particle size within the sediments. As recognized by ref. 22, there are large differences in population-level sediment reworking both within and among taxonomic groups, which also manifest in energy-transfer rates. Taxa with high reworking rates—e.g., benthic molluscs—produce energy fluxes comparable to those from cementation in sand-sized particles, even at high saturation states (*Ω* = 10). In contrast, macroepifauna such as whales rank as relatively inefficient; although large-bodied animals can rework large amounts of sediment over short timescales, their population reworking rates are suppressed by low population densities and infrequent interactions with the seafloor (22).

Rates of energy change for the interfacial free energy of sediments vary as functions of particle size and saturation state (Eq. **3**). Fine-grained particles have high reactive surface areas (*A*_*sp*_) per unit mass, and so crystal growth rates—and by extension, changes in interfacial energy—scale inversely with grain size under a fixed saturation state. Within discrete grain size categories (e.g., mud or fine sand), energy-transfer rates vary by approximately one order of magnitude as saturation states are varied from *Ω* = 3, the spatial average for the modern tropical surface seawater (42), to *Ω* = 10, which occurs in locally anoxic porewaters and has been hypothesized to represent higher saturation states in earlier Phanerozoic marine basins (43).

For physical energy transfer, endmember burrowing models produce different estimates for energy fluxes: the crack-propagation model predicts higher median rates of energy transfer than the excavation model. Although most taxa do not fall neatly into either crack propagation or excavation—many exhibit other behaviors entirely—previous studies provide important context for the most salient patterns in Fig. 2. As noted above, muddy carbonates are associated with high chemical energy fluxes because small particle sizes correspond to high reactive surface areas (35). These energy transfer rates apparently cannot be overcome by the lower endmember model, excavation. A key question, then, is which taxa burrow crack propagation, as their calculated energy fluxes are among the only ones that compete with cementation in muddy substrates. Macro-epifauna such as whales do not actually burrow per se, and it seems likely that their brief interactions with the substrate are akin to excavation. In contrast, recent experimental work shows that both polychaetes (bristle worms) and some bivalves use peristaltic motion to burrow by crack propagation (39), suggesting that the higher-end estimates of energy fluxes are plausible for these taxa.

### Before Bioturbation: Sediment Transport vs. Cementation.

**Fig. 3.**
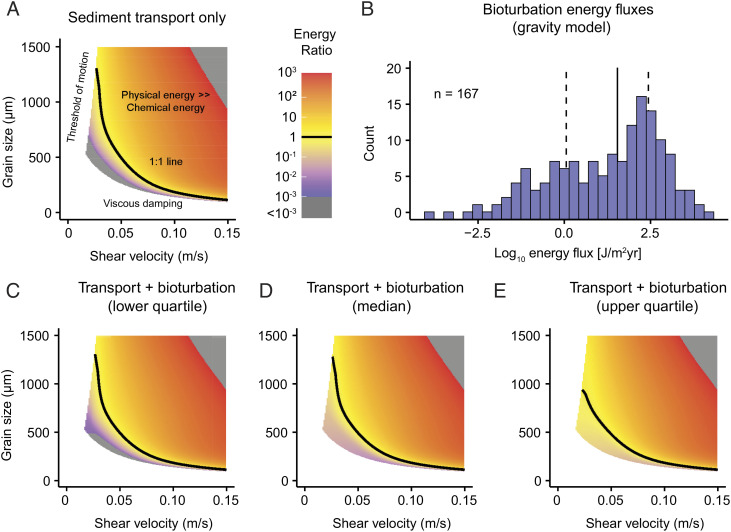
Full energy model combining cementation, sediment transport, and bioturbation. (*A*) Energy ratios, Eq. **8** as functions of shear velocity and grain size with no bioturbation. Note that grain size affects not only physical energy, Eq. **5** but also chemical energy, Eq. **3** because the surface area term depends on grain size. (*B*) Histogram showing energy fluxes calculated by the gravitational model, Eq. **9**. The solid line shows the median and the dashed lines show the lower and upper quartiles (35). Conditions that favor cementation (low energy ratios) occur near the edges of the plot: the left edge represents the threshold of particle motion, while the bottom edge represents viscous damping of grain impacts by the near-bed fluid. (*C*) Energy ratios with low animal bioturbation. The calculation is identical to that in *B*, except animals supply additional physical energy equal to the lower quartile in *A*. Although the plot is similar to *B*, even a small amount of bioturbation sets a lower bound on energy ratios near the threshold of motion and when viscous damping occurs. (*D*) Energy ratios with animal bioturbation equal to the median in *A*. The lower bound on energy ratios increases, but the position of the 1:1 line is similar to that in *B*. (*E*) Energy ratios with animal bioturbation equal to the upper quartile in *A*. The loss of energy ratios far below the 1:1 line suggests animals drastically alter energy ratios in environments where sediment transport does not transfer large amounts of energy to the bed.

Before the diversification and radiation of major animal groups in the early Paleozoic, bioturbation was either minimal or restricted to the uppermost sediment depths (24). Thus, in the Precambrian, sediment transport rather than animal activity transferred the majority of physical energy to the substratum. We depicted physical environments as bivariate plots of grain size and shear velocity (7). The contours in Fig. 3*A* depict the ratio between rates of kinetic energy change and chemical energy change:

[8] $$\begin{array}{c} \left(\frac{dE_{\mathrm{sed}}}{\mathrm{dt}}\right)/\left(\frac{dE_{\mathrm{chem}}}{\mathrm{dt}}\right), \end{array}$$

where red shading indicates sedimentary environments with kinetic energy budgets exceeding their chemical ones by at least two orders of magnitude. The boundary conditions of this model space are set by the threshold of particle motion, below which sediments are immobile on the bed surface, and by viscous damping forces, which dissipate the kinetic energy of flowing sediment particles in the near-bed fluid before it can be transferred to the substratum (Fig. 3). In the absence of bioturbation, potential chemical energy exceeds the competing physical energy, and cementation is favored near these boundaries. Note that grain size, shown on the vertical axis in Fig. 3*A*, affects both the physical energy flux in the numerator of Eq. **8** as well as the chemical energy flux in the denominator. The influence of grain size upon chemical energy reflects the inverse relationship between the diameter and surface area assumed in Eq. **4**.

### The Full Model: Bioturbation, Sediment Transport, and Cementation.

In the full model, both bioturbation and sediment transport act against cementation. Thus, energy-transfer rates in sedimentary depositional environments can be expressed as the ratio of physical energy fluxes (sediment transport and bioturbation) to cementation:

[9] $$\begin{array}{c} \left(\frac{dE_{\mathrm{sed}}}{\mathrm{dt}}+\frac{dE_{\mathrm{bio}}}{\mathrm{dt}}\right)/\left(\frac{dE_{\mathrm{chem}}}{\mathrm{dt}}\right). \end{array}$$

Under this scenario, bioturbation provides a “baseline” of physical energy that affects environments that were previously favorable for cementation because of low shear velocities and/or small sedimentary particle sizes (Fig. 3). Note that since modern rates of energy transfer from bioturbation vary over nine orders of magnitude, the overall effect of bioturbation depends on which taxa are present, and whether extant bioturbation rates presented by ref. 22 are applicable to ancient taxa. A simple way to approach the problem is to model different bioturbation scenarios defined by their relation to the distribution of burrowing rates in modern taxa. Adding low amounts of bioturbation to Eq. **9**—defined as the lower quartile of excavation rates calculated from modern taxa (Fig. 3*B*)—produced little discernible difference from the abiotic case (Fig. 3 *A* and *C*). However, adding energy-transfer rates from the median or upper quartile in Fig. 3*B* pushed energy ratios toward unity at the edges of the plot. Under higher rates of bioturbation, animals can reduce—or even outright eliminate—the portion of grain size–shear velocity space that is favorable for cemented substrates.

We note that the values for saturation state (*Ω*) and energy transfer from bioturbating organisms are highly variable throughout space and time, and so, Fig. 3*B* depicts a general scenario by modern chemistry, taxonomic diversity, and population densities. However, solutions in the form of Fig. 3 will show similar contour patterns, but the absolute values would shift by several orders of magnitude due to variations in either porewater chemistry or animal evolution and ecology. The low, medium, and high bioturbation scenarios presented in Fig. 3 *C* and *D* also provide some idea about how biological differences through space or time might affect seafloor environments. In fact, one way to interpret the progression from no bioturbation in Fig. 3*A* to median bioturbation in Fig. 3*C* is as a general trend from the Late Proterozoic to present. Another caveat is that the approach outlined here treats bioturbation, sediment transport, and chemistry as independent variables rather than interdependent ones. For example, the interplay between bioturbation rates and porewater chemistry via bioirrigation is a fundamental concept in both modern (30, 31) and ancient (32, 33, 44) marine environments. Nevertheless, the contour plots in Fig. 3 provide at least a first-order overview of potential interactions between physical, chemical, and biological variables.

## Discussion

Our model explains seafloor cohesion in terms of physical, chemical, and biological factors that can be measured directly in modern environments. To apply the model results to ancient sediments would require sedimentary observations with direct measurements such as grain size and sedimentary bedforms, which are readily available throughout the rock record. Grain size factors into the expressions for cementation, Eq. **3** and sediment transport, Eq. **5**; smaller grain sizes cement more quickly because they have high relative surface areas (35) and because of viscous damping as the physical energy transfer due to particle–bed collisions (Fig. 3). Flow velocities and frequencies of transport cannot be directly calculated but may be estimated from a combination of bedform stability diagrams (45) and transport frequency in modern environments (46). Thus, the physical environmental variables related to sediment transport can be constrained in many ancient strata.

In contrast, chemical and biological variables of interest need to be estimated from fossil data, proxy records, or Earth-systems models. The saturation state of carbonate minerals, which affects cementation rates, requires that at least two variables of the carbonate system are known (47). While estimates of atmospheric carbon dioxide levels have been made for the Phanerozoic (48), the search for geochemical proxies for marine pH (49) or alkalinity (50) beyond the Cenozoic has proven challenging. Models of these two terms can instead use indirect sedimentary proxies from carbonate facies (5, 51) but labor under large uncertainties in saturation states in the surface oceans and in porewaters. For bioturbation, its depth and intensity can be approximated from semiquantitative measures of sedimentary reworking and the density of trace fossils (52); additional relationships between trace fossils and burrower activity and population density are also possible (53).

Thus, one potential use of the model developed here is to place the disparate results from stratigraphic studies, Earth systems models, and geochemical proxy records into a common framework. We first interpreted a set of model results across broad spatial and temporal trends in Phanerozoic substrate mobility before focusing on the solution space for the physical, chemical, and biological changes across a singular, spectacular event: the End Permian Extinction.

### First-Order Spatial and Temporal Changes in Seafloor Cohesion.

Fine-grained environments should promote greater bed cohesion if animal activity is negligible (Figs. 2 and 3). Many sedimentary textures associated with cohesion develop in quiet water environments owing to infrequent and/or low-energy sediment transport. Thus, sediment cohesion tends to increase with bathymetry. This is often the case for textures such as flat-pebble conglomerates (23) and sea-floor fans (6, 57), which often form below fair-weather wave base where bed shear stress is low and sediment transport is intermittent. To a first order, the model results show that cohesion is inversely related to shear velocities and grain sizes, indicating that quiet-water environments are more prone to cementation Fig. 3.

Carbonate-cemented substrates have been observed in modern carbonate environments (60), but they are less common than in carbonates from the early Paleozoic, especially below storm weather wave base (23). The results in Figs. 2 and 3 have several useful implications for interpreting the sedimentary record of seafloor cohesion across space and time. In the absence of significant sediment transport (Fig. 2*A*), the range of energy transfer rates from modern organisms overlaps substantially with the range for cementation across a variety of particle sizes and cementation rates. Because energy fluxes from bioturbation span ten orders of magnitude, the local ecology of organisms is a major control—perhaps even the dominant one—on whether or not carbonate cementation occurs in low transport settings. In turn, secular changes in the abundance of carbonate facies may dominantly reflect underlying variables that affect population reworking rates such as biomass density, behavior, and body size.

### Substrate Cohesion and the End-Permian Extinction.

Changes in substrate cohesion are commonly associated with intervals of drastic gains or losses in marine biodiversity, as exemplified in the End-Permian and Early Triassic. In addition to biodiversity loss, the End-Permian and Early Triassic are associated with large carbon cycle perturbations and ocean anoxia, which likely also resulted in substantial changes to the carbonate chemistry of seawater (61). This confluence of factors raises an interesting question: does the occurrence of cohesive carbonate substrates correspond to carbon cycle changes and ocean anoxia—the root causes of the extinction—or do they instead represent the effects of the extinction by signaling major loss of animal biomass in addition to biodiversity loss?

We considered quiet-water environments where the ratio of physical-to-chemical energy fluxes is approximately:

[10] $$\begin{array}{c} F=\left(\frac{dE_{\mathrm{bio}}}{\mathrm{dt}}\right)/\left(\frac{dE_{\mathrm{chem}}}{\mathrm{dt}}\right). \end{array}$$

For geologic purposes, it is helpful to consider an additional ratio, *ΔF*, representing the change in physical-to-chemical energy fluxes from one point in time to another:

[11] $$\begin{array}{c} \Delta F=\frac{F_{t_{2}}}{F_{t_{1}}}. \end{array}$$

Eq. **11** provides a simple way to visualize the sensitivity of the model to changes in carbonate chemistry—either bulk seawater or within anoxic porewaters—against changes in total animal biomass and body size, which is fully described in *SI Appendix*. Using previous estimates of saturation state from climate modeling (58) and from the occurrence of giant ooids (55), the model shows that these processes could produce an order-of-magnitude change in energy fluxes favoring more stable substrates. However, a similar calculation using proposed losses in animal biomass (62) could produce nearly the same response. While these factors are not mutually exclusive—they may have operated together—they do operate over different timescales and make different predictions in terms of the system response. For example, increases in alkalinity due to silicate weathering feedbacks produce strong responses but should be localized to 100s of ky after carbon isotope excursions (63). In contrast, biomass losses after the end-Permian likely persisted for millions of years based on the abundance of skeletal grains in carbonates (62) and a paucity of burrows in Early Triassic strata (64). Given the long timescales involved, it seems likely that protracted occurrences of “anachronistic” carbonate facies (57) simply represent the long timescale of ecological recovery rather than repeated short-term perturbations to the ocean–atmosphere system.

**Fig. 4.**
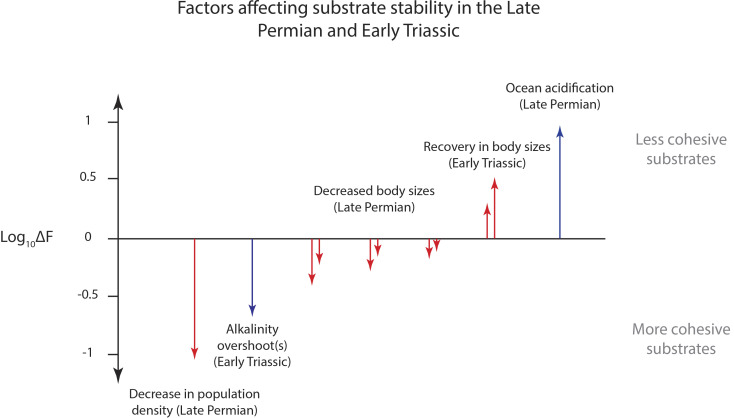
Modeled effects of the end-Permian event on marine substrate stability in Early Triassic environments. The vertical axis shows changes in physical-to-chemical energy fluxes between two arbitrary points in time, Eq. **11**. The length and direction of each vector represent the result of adjusting a single parameter in the model based on previous findings in the literature. Chemical and biological factors that might have enhanced substrate stability include an overall loss of animal biomass as suggested by reduced volumes of shelly material in carbonates (54), an increase (or “overshoot”) in ocean alkalinity due to increased continental weathering (55), or a reduction in animal body sizes without an assumed reduction in the number of individuals (56). Note that both chemical and biological mechanisms are the same order of magnitude and present valid explanations for the appearance of ‘anomalous’ carbonate features in the Early Triassic (57). In contrast, periods of higher substrate mobility might correspond to acidification of the surface ocean and reduced saturation states for carbonate minerals (58) and later a recovery in animal body sizes (59). A detailed explanation of scaling relationships and previous studies are provided in *SI Appendix*.

The rough equivalence between biological and chemical influences on substrate stability—as calculated from both modern data (Fig. 2) and for the End Permian example (Fig. 4)—highlights that animals play an even larger role than previously realized in shaping marine environments and their sedimentary records. Given the results in Fig. 3, low-energy environments without animals are prone to cementation feedbacks: cements bind grains together, which creates larger effective particle sizes, rendering sediment even harder to move. The situation is analogous to terrestrial systems where energy-based frameworks have highlighted the role of animals in landscape evolution and sediment transport (65, 66). The parallel approach developed here provides a new tool for relating sedimentary strata to time-varying evolutionary and ecological factors which are traditionally difficult to measure in the rock record.

#### SI datasets.

The dataset used to generate Fig. 2 is available as Supplementary Dataset S1.

## Materials and Methods

### Energy Associated with Crack Propagation.

Experimental work by ref. 38 shows that burrowing in cohesive substrates can be modeled as propagating a fracture tip. Under these conditions, the work done on the substrate is

[12] $$\begin{array}{c} E_{\mathrm{cr}}=G_{c}wz, \end{array}$$

where *E*_*cr*_ is the work [J] required to propagate the crack, *z* is the distance [L] the crack propagates, *G*_*c*_ [J] [L^−2^] is the fracture toughness, and w [L] is the width of the crack, which is assumed to be equal to the diameter of the burrow.

If the fracture tip propagates at some linear velocity $\frac{\mathrm{dz}}{\mathrm{dt}}$, then the rate of working on the sediments is

[13] $$\begin{array}{c} \frac{dE_{\mathrm{cr}}}{\mathrm{dt}}=G_{c}w\frac{\mathrm{dz}}{\mathrm{dt}}. \end{array}$$

Further interpretation of Eq. **13** requires information about how burrowing rates are measured and reported in the literature. The values used here were reported by ref. 22 as a volume of sediment [L^3^] reworked by a population of organisms with a certain density at or below the seafloor [individuals] [L^−2^]. This quantity, which we refer to as the volumetric reworking rate *R*, has units of [L^3^] [L^−2^] [t^−1^].

To adapt Eq. **13** to the volumetric reworking rate R, we need information regarding body size, specifically the cross-sectional area of the organism which sets the burrow width w. A common method is to approximate an animal’s body by fitting an ellipsoid with a circular cross-section (59). The relationship between the linear burrowing rate $\frac{\mathrm{dz}}{\mathrm{dt}}$ and the volumetric reworking rate R is then

[14] $$\begin{array}{c} \frac{\mathrm{dz}}{\mathrm{dt}}=\frac{R}{\pi b^{2}}, \end{array}$$

where the burrow radius, *b* is half the burrow width *w* in Eq. **13**. Combining Eqs. **13** and **14** casts $\frac{dE_{\mathrm{cr}}}{\mathrm{dt}}$ in terms of the volumetric reworking rate *R*:

[15] $$\begin{array}{c} \frac{dE_{\mathrm{cr}}}{\mathrm{dt}}=\frac{2G_{c}R}{\pi b}. \end{array}$$

Dimensional analysis of Eq. **15** shows that $\frac{dE_{\mathrm{cr}}}{\mathrm{dt}}$ has units of [J] [L^−2^] [t^−1^]. These units represent a rate of energy transfer per unit surface area, which we interpret as the rate at which animals transfer energy across the sediment–water interface.

### Energy Associated with Excavation.

We now consider an alternate estimate of energy associated with burrowing. Consider a cylindrical burrow perpendicular to the sediment–water interface in which the sediment is excavated from the substrate and redeposited at the sediment–water interface. At a minimum, burrow excavation does work against gravity by lifting particles toward the sediment–water interface:

[16] $$\begin{array}{c} E_{\mathrm{gr}}=mgz, \end{array}$$

where E_*gr*_ is the change in gravitational energy [J], *m* is the mass of sediment moved [M], *g* is the gravitational acceleration [L] [t^−2^], and *z* is the vertical distance [L]. The (buoyant?) mass of some portion of the cylindrical burrow with a vertical thickness of *Δ*z is

[17] $$\begin{array}{c} m=\left(1-\phi \right)\left(\rho_{s}-\rho_{w}\right)\pi b^{2}\Delta z, \end{array}$$

where *ϕ* is the porosity of the sediment, *ρ*_*s*_ and *ρ*_*w*_ are the densities of sediment and water, respectively, b is the burrow radius, and z is the distance from the center-of-mass to the sediment–water interface. The work of lifting n segments is

[18] $$\begin{array}{c} \sum_{i=1}^{n}E_{\mathrm{gr}}=\sum_{i=1}^{n}\left[\left(1-\phi \right)\left(\rho_{s}-\rho_{w}\right)\pi b^{2}\Delta z\right]gz_{i}. \end{array}$$

Taking the limit as n goes to infinity and *Δ*z goes to zero, the work for excavating a cylindrical burrow that extends to some depth d below the sediment–water interface is

[19] $$\begin{array}{c} \begin{array}{cc} E_{\mathrm{gr}} & =\int_{0}^{d}\left[\left(1-\phi \right)\left(\rho_{s}-\rho_{w}\right)\pi b^{2}dz\right]gz\\ & =\frac{1}{2}\left(1-\phi \right)\left(\rho_{s}-\rho_{w}\right)\pi b^{2}gd^{2}. \end{array} \end{array}$$

The result in Eq. **19** is the same as if we had used the entire mass of the burrow in Eq. **17** and raised the center-of-mass from half the burrow depth to the surface.

We now consider the work done in excavating *n* identical burrows:

[20] $$\begin{array}{c} E_{\mathrm{gr}}=n\left[\frac{1}{2}\left(1-\phi \right)\left(\rho_{s}-\rho_{w}\right)\pi b^{2}gd^{2}\right] \end{array}$$

If we approximate the rate at which new burrows are created as a continuous process, then

[21] $$\begin{array}{c} \frac{E_{\mathrm{gr}}}{\mathrm{dt}}=\frac{\mathrm{dn}}{\mathrm{dt}}\left[\frac{1}{2}\left(1-\phi \right)\left(\rho_{s}-\rho_{w}\right)\pi b^{2}gd^{2}\right]. \end{array}$$

Under this approximation, the rate at which new burrows are created is related to the linear burrowing velocity by

[22] $$\begin{array}{c} \frac{\mathrm{dn}}{\mathrm{dt}}=\frac{1}{d}\frac{\mathrm{dz}}{\mathrm{dt}}. \end{array}$$

Using Eq. **14**, the relationship to the volumetric burrowing rate reported by (22) is

[23] $$\begin{array}{c} \frac{\mathrm{dn}}{\mathrm{dt}}=\frac{R}{\pi b^{2}d}. \end{array}$$

Substituting Eq. **23** into Eq. **21**, we arrive at

[24] $$\begin{array}{c} \frac{dE_{\mathrm{gr}}}{\mathrm{dt}}=\frac{1}{2}\left(1-\phi \right)\left(\rho_{s}-\rho_{w}\right)Rgd. \end{array}$$

Dimensional analysis shows that Eq. **24** has units of [J] [L^−2^] [t^−1^]. The interpretation of these units is the same as for Eq. **15** in the preceding section: a rate of energy transfer per unit area of the sea floor.

### Energy Associated with Crystal–Fluid Interfaces.

Consider the expressions for interfacial energy between sediments and porewater (15):

[25] $$\begin{array}{c} \begin{array}{cc} G_{\mathrm{int}}= & \sigma A\\ \frac{dG_{\mathrm{int}}}{\mathrm{dt}}= & \sigma \frac{\mathrm{dA}}{\mathrm{dt}}, \end{array} \end{array}$$

where *σ* is a constant representing the specific free energy for the mineral–fluid contact [J] [L^−2^] and *A* is the crystal surface area [L^2^]. To derive **1**, we need an expression for $\frac{\mathrm{dA}}{\mathrm{dt}}$, the rate at which the surface area is changing within sediments. Following (67), the relationship between surface area and the porosity can be approximated by

[26] $$\begin{array}{c} A=A_{0}{\left(\frac{\phi }{\phi_{0}}\right)}^{2/3}, \end{array}$$

where A_0_ and *ϕ*_0_ are the initial surface area and porosity of sediments prior to cementation. Taking the time derivative yields:

[27] $$\begin{array}{c} \frac{\mathrm{dA}}{\mathrm{dt}}=\frac{2}{3}\frac{A_{0}}{\phi_{0}}{\left(\frac{\phi }{\phi_{0}}\right)}^{-1/3}\frac{d\phi }{\mathrm{dt}}. \end{array}$$

It is convenient to rewrite the porosity *ϕ* and its derivative in terms of the volume of carbonate, V_*c*_, which includes the original sediment plus diagenetic precipitates:

[28] $$\begin{array}{c} \begin{array}{cc} \phi & =\left(1-\frac{V_{c}}{V_{t}}\right)\\ \frac{d\phi }{\mathrm{dt}} & =-\frac{1}{V_{t}}\frac{dV_{c}}{\mathrm{dt}}. \end{array} \end{array}$$

For the derivative $\frac{dV_{c}}{\mathrm{dt}}$, we use an expression that describes carbonate precipitation for grains such as ooids (5):

[29] $$\begin{array}{c} \frac{dV_{c}}{\mathrm{dt}}=k{\left(\Omega -1\right)}^{n}\frac{M_{c}}{\rho_{c}}A. \end{array}$$

Combining Eqs. **26**, **27**, **28** and **29** yields

[30] $$\begin{array}{c} \frac{\mathrm{dA}}{\mathrm{dt}}=-\frac{2}{3}\frac{A_{0}^{2}}{\phi_{0}V_{t}}{\left[\frac{V_{t}-V_{c}}{V_{t}-V_{c0}}\right]}^{1/3}\frac{M_{c}}{\rho_{c}}k{\left(\Omega -1\right)}^{n}. \end{array}$$

Here, the negative sign denotes the inverse relationship between the total volume of carbonate and the surface area of the pores. The term in square brackets takes on values between zero and one. When V_*c*_ = V_*c*0_—at the hypothetical moment that cementation begins—the term in square brackets is equal to one and the magnitude of $\frac{\mathrm{dA}}{\mathrm{dt}}$ is maximized. In contrast, as V_*c*_ approaches the total volume, V_*c*0_, then $\frac{\mathrm{dA}}{\mathrm{dt}}$ goes to zero.

If we consider the case where V_*c*_ = V_*c*0_, then combining Eqs. **1** and **30** yields the maximum rate for change in interfacial energy:

[31] $$\begin{array}{c} \frac{\mathrm{dE}}{\mathrm{dt}}=-\frac{2}{3}\sigma \frac{A_{0}^{2}}{\phi_{0}V_{t}}\frac{M_{c}}{\rho_{c}}k{\left(\Omega -1\right)}^{n}. \end{array}$$

Dimensional analysis shows that Eq. **31** has units of [J] [t^−1^].

The reactive surface area of sediments is often reported as a specific surface area with units of [L^2^] [M^−1^]. The initial surface area within an arbitrary volume containing sediment and fluid beneath is

[32] $$\begin{array}{c} A_{0}=A_{\mathrm{sp}}\left(1-\phi_{0}\right)V_{t}\rho_{c}. \end{array}$$

Combining Eqs. **31** and **32** yields

[33] $$\begin{array}{c} \frac{\mathrm{dE}}{\mathrm{dt}}=-\frac{2}{3}\sigma \frac{{\left(A_{\mathrm{sp}}\left(1-\phi_{0}\right)\right)}^{2}}{\phi_{0}}\rho_{c}M_{c}k{\left(\Omega -1\right)}^{n}V_{t}. \end{array}$$

As before, the units of Eq. **33** has units of [J] [t^−1^]. Thus, the equation is not yet normalized per unit area of the seafloor unlike the values for bioturbation given in the preceding sections. To convert the units in Eq. **33**, we envision a rectangular prism below the seafloor with one side at the sediment–water interface. If we divide Eq. **33** by V_*t*_, we get the volume-normalized energy change in units of [J] [L^−3^] [t^−1^]:

[34] $$\begin{array}{c} \frac{\mathrm{dE}}{\mathrm{dt}}=-\frac{2}{3}\sigma \frac{{\left(A_{\mathrm{sp}}\left(1-\phi_{0}\right)\right)}^{2}}{\phi_{0}}\rho_{c}M_{c}k{\left(\Omega -1\right)}^{n}. \end{array}$$

To convert Eq. **34** into units of [J] [L^−2^] [t^−1^], we multiply by a vertical length scale, L. To facilitate the comparisons in the main text, we use a length scale of 0.05 m, which is the median burrow depth reported by ref. 22. The result yields Eq. **3** in the main text.

## Supplementary Material

Appendix 01 (PDF)

Dataset S01 (XLSX)

## Data Availability

All study data are included in the article and/or *SI Appendix*. Previously published data were used for this work; Thayer (1983). The scripts used to compile Figs. 2 and 3 are publicly available at https://doi.org/10.5281/zenodo.7430472.
