# Supplementary material for: Tracing energy inputs into the seafloor using carbonate sediments
Source: Proc Natl Acad Sci U S A. 2023 Feb 21;120(9):e2215833120. doi: 10.1073/pnas.2215833120 (PMC9992785; doi:10.1073/pnas.2215833120)
Supplement: Supplementary file 1 — Appendix 01 (PDF) [file pnas.2215833120.sapp.pdf]

Supporting Information for

Tracing energy inputs into the seafloor using carbonate sediments

BP Smith, SM Edie and WW Fischer

bpsmith@caltech.edu

This PDF file includes:

Supporting text
Table S1
SI References

Supporting Information Text

A. Scaling relationships and the End Permian Mass Extinction. One use of the model is to place research into biological and chemical changes during mass extinctions into a quantitative framework. Notably, values from the literature can produce order-of-magnitude estimates of Earth-system responses without having to fully solve the model. Below we outline three cases pertaining to the end-Permian extinction: changes in bioturbation via reduced population densities, changes in biomass density brought about by reduced body sizes, and changes in substrate stability brought on by changes in the saturation state of seawater with respect to carbonate minerals.

First, we consider the ratio of physical to chemical energy fluxes near the sediment-water interface:

$$F = \left(\frac{dE_{sed}}{dt} + \frac{dE_{bio}}{dt}\right) / \left(\frac{dE_{chem}}{dt}\right) \quad [1]$$

where E has units of [J][L^{-2}][t^{-1}] and the subscripts denote fluxes of energy from sediment transport, bioturbation, and cementation. Eqn. **??** is identical to Eqn. 8 in the main text.

We now consider two energy ratios that represent substrate conditions at some time before (t_1) and some time after (t_2) an event. The relative change in energy fluxes is:

$$\Delta F = \frac{F_{t_2}}{F_{t_1}} \quad [2]$$

Eqn. 2 can be simplified for cases where only a single variable changes with time while the others are held constant. If chemical conditions do not change, then quiet-water environments ($dE_{sed}/dt \approx 0$) are defined by:

$$\Delta F = \left(\left.\frac{dE_{bio}}{dt}\right|_{t_2}\right) / \left(\left.\frac{dE_{bio}}{dt}\right|_{t_1}\right) \quad [3]$$

In contrast, if chemical conditions change but physical energy from animals and sediment transport remains constant:

$$\Delta F = \left(\left.\frac{dE_{chem}}{dt}\right|_{t_1}\right) / \left(\left.\frac{dE_{chem}}{dt}\right|_{t_2}\right) \quad [4]$$

B. Changes in population density. Consider a case where changing population densities affect substrate stability. We assume the population reworking rate, R, depicted in Eqns. 5 and 6 in the main text is linearly proportional to the population density. Under this assumptions, the order-of-magnitude effect on substrate stability has a simple interpretation in quiet-water environments by Eqn. 3. For the End Permian, ref. (1) counted shelly fauna in carbonates across the Permian-Triassic boundary and estimated that biovolume decreased by 1 order an order of magnitude. If the decrease in shelly fauana represents decreasing population densities, then:

$$log_{10}(\Delta F) = log_{10}(F|_{t_2}) - log_{10}(F|_{t_1}) = -1 \quad [5]$$

Note that this result does not depend on assumptions about crack propagation versus excavation endmembers since both Eqns. 5 and 6 in the main text are linearly proportional to the population reworking rate, R.

C. Changes in body size. In addition to reduced population densities, the End Permian mass extinction also corresponded with a decrease in body size for many marine taxa (2, 3). We now consider another endmember case where body sizes change but population densities are constant. Unlike the previous case, the scaling here is sensitive to crack propagation versus excavation mechanisms. Re-arranging Eqn. 11 in the main text shows emphasizes the relationships among body size, linear burrowing velocities, and volumetric reworking rates:

$$R = \pi b^2 \frac{dz}{dt} \quad [6]$$

where R is the volumetric reworking rate, b is the radius of the organism's circular cross section, and dz/dt is the linear burrowing velocity. If dz/dt is not a function of body size, then substituting Eqn. 6 in the main text into Eqn. 3 yields:

$$\Delta F = \frac{2G_c b_2 \frac{dz}{dt}}{2G_c b_1 \frac{dz}{dt}} = \frac{b_2}{b_1} \quad [7]$$

where b_1 and b_2 represent burrow radii at times t_1 and t_2.

By similar logic, substituting Eqn. 18 into Eqn. 3 yields the following expression for excavation:

$$\Delta F = \frac{\frac{dn}{dt}\left[\frac{1}{2}(1-\phi)(\rho_s - \rho_w)\pi b_2^2 g d^2\right]}{\frac{dn}{dt}\left[\frac{1}{2}(1-\phi)(\rho_s - \rho_w)\pi b_1^2 g d^2\right]} = \frac{b_2^2}{b_1^2} \quad [8]$$

For further analysis, we assumed that animal body volume can be approximated as ellipsoids with a circular cross sections following ref. (3):

$$V = \frac{4}{3}\pi ab^2 \tag{9}$$

where V is the volume, b is the maximum radius of their circular cross-section—assumed to be interchangeable with the radius of the burrow—and a is the long dimension. If changes in body volume maintain a constant aspect ratio among ellipsoids, i.e., the scaling among axes follows:

$$\frac{b_1}{a_1} = \frac{b_2}{a_2} = k \tag{10}$$

where k is some constant, then:

$$V = \frac{4}{3}\pi kb^3 \tag{11}$$

By Eqn. 7, the relative change in energy fluxes for crack propagation scale with body size raised to the one-third:

$$\Delta F = \frac{b_2}{b_1} = \left(\frac{V_2}{V_1}\right)^{1/3} \tag{12}$$

By Eqn. 8,

$$\Delta F = \frac{b_2^2}{b_1^2} = \left(\frac{V_2}{V_1}\right)^{2/3} \tag{13}$$

In both cases, the exponents are consistent with dimensional analysis of Eqn. 10 and Eqn. 18 in the main text. The expression for crack propagation (Eqn. 10) is proportional to the width of the burrow in units of [L], so we expect that the scaling between body volume [L^3] and burrow width is on the order of $V^{1/3}$. Similarly, Eqn. 18 depends on the cross-sectional area of the burrow, which has units of [L^2], so we expect that the scaling with body volume is on the order of $V^{2/3}$.

We acknowledge that the cases outlined above are highly simplified, and that animals use a wide array of burrowing mechanisms to disrupt sediment. However, it seems likely that most mechanisms either depend on the width of the burrow and thus exhibit $[L] \sim V^{1/3}$ scaling or else depend on cross-sectional area and exhibit $[L^2] \sim V^{1/3}$ scaling. In a population of animals exhibiting complex behaviors, we can consider the cases outlined above as reasonable endmembers that bracket the result:

$$\left(\frac{V_2}{V_1}\right)^{1/3} \leq \Delta F \leq \left(\frac{V_2}{V_1}\right)^{2/3} \tag{14}$$

D. Changes in saturation states of carbonate minerals. We now consider a case where energy expanded by animals is constant, but chemical conditions change. By Eqn. 4 and Eqn. 3 in the main text:

$$\Delta F = \frac{-\frac{2}{3}\sigma\frac{(A_{sp}(1-\phi_0))^2}{\phi_0}\rho_c M_c k(\Omega_1 - 1)^n * L}{-\frac{2}{3}\sigma\frac{(A_{sp}(1-\phi_0))^2}{\phi_0}\rho_c M_c k(\Omega_2 - 1)^n * L} = \frac{(\Omega_1 - 1)^n}{(\Omega_2 - 1)^n} \tag{15}$$

or:

$$log_{10}\Delta F = n(log_{10}(\Omega_1 - 1) - log_{10}(\Omega_2 - 1)) \tag{16}$$

Eqn. 16 states that relative changes in chemical energy fluxes mostly depend on the power law exponent, n, and on the relative difference in saturation states. For the Late Permian and Early Triassic, Eqn. 16 can be applied to substrate stability either to estimate the effects of ocean acidification or to estimate the effects of an alkalinity "overshoot" in the Early Triassic due to enhanced silicate weathering (4, 5). A third (very plausible) explanation for unusual Triassic carbonates is that anoxic conditions persisted in some marine environments and in porewaters long after the extinction (6). However, it is difficult to treat such a scenario in the simple approaches outlined; given the close relationship between bioturbation and porewater chemistry (7, 8), it seems very likely that biomass loss and anoxia are linked, and thus it makes little sense to treat them as independent variables even in a thought experiment.

E. Applications to the End Permian. The scaling relationships presented in the preceding sections offer a convenient way to estimate order-or-magnitude effects on substrate stability from a wide variety of sources. A selection of mechanisms, studies, and estimated variables are presented in Table S1. The vector lengths and directions presented in Fig. 4 in the main text correspond to the column labeled $log_{10}\Delta F$.

Table S1. Sources and energy scaling for Figure 4

proxy/model	interpretation	scaling	$\log_{10}(\Delta F)$	source	notes
skeletal abundance	population density	Eqn. 5	-1	ref. (9)	Estimate based on -1 order of magnitude change in skeletal versus non-skeletal carbonates from the Great Bank of Guizhou.
shell size	reduced body size	Eqn. 14	from -0.18 to -0.37	ref. (2)	Estimate based on a median 0.55 order of magnitude change in gastropod shell volume across the P-T. Measurement compiled from global P-T sections and represents the lowest stratigraphic resolution in Table 1 in the reference.
body size	reduced body size	Eqn. 14	from -0.07 to -0.13	ref. (3)	Estimated from a 0.2 order-of-magnitude change in marine animal body size in the Late Permian (their Fig. 1)
burrow width	reduced body size	Eqns. 12 and 13	from -0.12 to -0.23	ref/ (10)	Estimated from a change in *Planolites* burrow widths from 6.0mm in the Late Permian to 4.6mm in the Early Triassic
carbon cycle model	ocean acidification	Eqn. 15	0.9	ref (11)	Estimated from model results suggesting the aragonite saturation state of the surface ocean dropped from $\Omega = 6$ to $\Omega = 3$ across the P-T boundary.
body size	increased body size	Eqn. 14	0.23-0.46	ref. (3)	Estimated from a 0.7 order-of-magnitude recovery in marine animal body size in the Early Triassic (their Fig. 1)
ooid sizes	increased saturation states	Eqn. 15	0.62	ref (5)	Based on saturation states inverted from ooid diameters. Uses a change in median long axis dimensions from 2mm to 4mm. The resulting saturation states depend on the fraction of time ooids spend in transport versus stationary on the bed (f), which is 0.1 to 0.25 for modern ooid-forming environments. Here we use f = 0.1, although Eqn. 15 does not appear sensitive to the ranges of f presented in ref (5)

References

1. JL Payne, DJ Lehrmann, J Wei, AH Knoll, The pattern and timing of biotic recovery from the end-Permian extinction on the Great Bank of Guizhou, Guizhou Province, China. *Palaios* **21**, 63–85 (2006).
2. JL Payne, Evolutionary dynamics of gastropod size across the end-permian extinction and through the triassic recovery interval. *Paleobiology* **31**, 269–290 (2005).
3. NA Heim, ML Knope, EK Schaal, SC Wang, JL Payne, Cope's rule in the evolution of marine animals. *Science* **347**, 867–870 (2015).
4. JL Payne, LR Kump, Evidence for recurrent early triassic massive volcanism from quantitative interpretation of carbon isotope fluctuations. *Earth Planet. Sci. Lett.* **256**, 264–277 (2007).
5. X Li, et al., Implications of giant ooids for the carbonate chemistry of Early Triassic seawater. *Geology* **49**, 156–161 (2021).
6. AD Woods, Assessing early triassic paleoceanographic conditions via unusual sedimentary fabrics and features. *Earth-Science Rev.* **137**, 6–18 (2014).
7. RC Aller, The effects of macrobenthos on chemical properties of marine sediment and overlying water in *Animal-sediment relations*. (Springer), pp. 53–102 (1982).
8. RC Aller, Bioturbation and remineralization of sedimentary organic matter: effects of redox oscillation. *Chem. Geol.* **114**, 331–345 (1994).
9. J Payne, S Finnegan, Controls on marine animal biomass through geological time. *Geobiology* **4**, 1–10 (2006).
10. M Luo, G Shi, LA Buatois, ZQ Chen, Trace fossils as proxy for biotic recovery after the end-Permian mass extinction: A critical review. *Earth-Science Rev.* **203**, 103059 (2020).
11. Y Cui, M Li, EE Van Soelen, F Peterse, WM Kürschner, Massive and rapid predominantly volcanic co_2 emission during the end-Permian mass extinction. *Proc. Natl. Acad. Sci.* **118**, e2014701118 (2021).
